# Open wavefront ablation terminates rotor-driven atrial fibrillation

**DOI:** 10.3389/fnetp.2026.1940428

**Published:** 2026-10-08

**Authors:** Arthur S. Bezerra, Robin Van Den Abeele, Bram Van Landeghem, Bjorn Verstraeten, Sebastiaan Lootens, Viktor Van Nieuwenhuize, Sander Hendrickx, Vincent F. M. Segers, Mattias Duytschaever, Nele Vandersickel

**Affiliations:** 1 Department of Physics and Astronomy, Ghent University, Ghent, Belgium; 2 Department of Pharmaceutical Sciences, Antwerp University, Antwerp, Belgium; 3 AZ Sint-Jan, Cardiology Department, Bruges, Belgium

**Keywords:** ablation, atrial fibrillation, simulations, topology, wavefronts

## Abstract

**Introduction:**

Atrial arrhythmias, notably atrial fibrillation (AF), exhibit complex spatiotemporal patterns that remain incompletely characterized. Based on topological constraints requiring counter-rotating reentries in two-dimensional manifolds, we present a framework classifying all atrial wavefronts into two fundamental archetypes: open and closed waves. Open waves are line-like structures bounded by endpoints that tend to self-perpetuate; closed waves are ring-like structures lacking endpoints that tend toward self-termination.

**Methods and Results:**

We demonstrate that complex wavefront interactions reduce to five elementary processes: (1) Open wave creation, (2) Open wave annihilation, (3) Closed wave creation, (4) Closed wave annihilation and (5) Open wave collision. Validation within a cohort of 600 patient-specific AF simulations showed that all observed events adhered to these constructed topological rules, suggesting that complexity of fibrillatory conduction within the studied computational framework arises from simple underlying principles. Furthermore, closing all open waves, achieved a 100% success rate when electrical silence was used as the primary criteria for efficacy.

**Discussion:**

This unified framework reconciles existing electrophysiological theories, including the multiple wavelet hypothesis, the mother rotor hypothesis, meandering rotors, and focal sources, while providing a foundation for optimizing clinical interventions.

## 1 Introduction

Haissaguerre et al. (1998) demonstrated that paroxysmal atrial fibrillation (AF) is triggered by irregular ectopic activity originating from the pulmonary veins and showed that pulmonary vein isolation (PVI) is an effective treatment. Conversely, the mechanisms sustaining persistent AF remain incompletely understood. Several hypotheses have been proposed. (1) The multiple wavelet theory posits that AF is maintained by continuously shifting reentrant wavelets that vary in location, size, shape, and number (Moe et al., 1964; Allessie, 1985; Child et al., 2018). (2) The mother rotor hypothesis suggests that a single, rapidly rotating reentry source generates wavebreaks in the surrounding tissue, giving rise to fibrillatory conduction patterns that resemble multiple wavelets (Jalife et al., 2002; 1998). (3) Closely related, the multiple rotor hypothesis attributes AF maintenance to several stable or meandering rotors distributed throughout the atria (Narayan et al., 2012; 2014; Morgan et al., 2016; Vigmond et al., 2016; Haissaguerre et al., 2014; Bayer et al., 2016; Zahid et al., 2016). Notably, these theories primarily assume AF dynamics occur within a single atrial layer. In contrast, two additional hypotheses explicitly invoke three-dimensional atrial structure. (4) The double-layer hypothesis proposes that persistent AF arises from electrical dissociation between the atrial endocardial and epicardial layers, with fibrillatory activity maintained by intermittent breakthroughs at residual connections (Allessie et al., 2010; de Groot et al., 2016; 2010; Gharaviri et al., 2020). (5) Finally, the micro-anatomic intramural reentry hypothesis suggests that AF may be driven by stable intramural reentrant circuits anchored within the atrial wall, particularly involving the pectinate muscle bundles (Hansen et al., 2015; Zhao et al., 2017).

In previous work, we demonstrated that reentries in regular atrial tachycardia (AT) are, in fact, paired topological objects that rotate around two critical boundaries (CBs) (Duytschaever et al., 2024; Van Den Abeele et al., 2025; Duytschaever et al., 2025). These boundaries are stable and correspond mostly to natural atrial openings such as the left pulmonary veins (LPV), right pulmonary veins (RPV), and mitral valve (MV) in the left atrium (LA), or the tricuspid valve (TV), inferior vena cava (IVC), and superior vena cava (SVC) in the right atrium (RA). In addition, any region of non-conducting tissue serves as an additional boundary, such as scar or previous ablation lesions.

This pairing arises from an index theorem, which states that the total topological charge (or index) associated with rotational activity over a closed surface must sum to zero (Davidsen et al., 2004; Vandersickel et al., 2024). A local topological charge (TC) can be computed around each boundary, and in practice one typically identifies one CB with index −1 and a second CB with index +1. Usually, one CB hosts a complete reentry, while another CB hosts an incomplete reentry (Duytschaever et al., 2024; 2025). Ablation that connects these two boundaries terminates the AT. If they remain disconnected, the tachycardia persists, either at the same or at a longer cycle length.

Subsequently, we extended this methodology to a computational model of AF and demonstrated that even in highly unstable arrhythmias, reentrant activity necessarily occurs in oppositely rotating pairs. These rotations may either be located around functional boundaries or anchored to anatomical boundaries (Bezerra et al., 2025). In this setting, however, connecting opposite wavefronts with a straight line terminated the arrhythmia in only 50% of cases, a proportion that increased to 90% when the connection followed the trajectory of the wavefront. Yet, some cases still persist. In this work, we explain why connecting opposite rotational pairs in the presence of meandering rotors does not always terminate the arrhythmia.

To this end, we shift our perspective from (critical) boundaries to waves and demonstrate that, for any arrhythmia in which the atrium can be represented as a two-dimensional surface, only two fundamental wave types exist: open waves and closed waves. This terminology was introduced only once, by Wiener and Rosenblueth (1946), and only in the context of atrial flutter. Furthermore, we will show that all other apparent wave types result from interactions between open and closed waves and are intrinsically transient, persisting only for brief interaction intervals.

Additionally, recent work has introduced a novel approach for terminating rotational activity using precisely timed electrical stimuli (DeTal et al., 2022). In this study, the authors demonstrate that appropriately timed stimuli can effectively “teleport” the endpoints of spiral waves to each other’s locations. This causes the two endpoints to annihilate, creating a single wavefront without endpoints and thereby terminating the rotational activity.

Building on these findings, this work establishes a novel theoretical framework based on the hypothesis that only two fundamental wave types exist: open and closed waves. Within this framework, we show that only five distinct types of transient interactions can occur between open waves, closed waves, and anatomical or functional obstacles: (1) open wave creation accompanied by closed wave annihilation, (2) open wave creation, (3) open wave annihilation, (4) open wave annihilation accompanied by closed wave creation, and (5) closed wave annihilation. We further demonstrate that the index theorem is universally satisfied within this framework. Finally, we use these principles to design a topology-based ablation strategy that reliably terminates rotor-driven atrial fibrillation in simulations by converting open waves into closed waves.

## 2 Theoretical framework

### 2.1 Open and closed waves

Throughout this work, we are working with single-atrium meshes, where each atrium is modeled as a two-dimensional compact manifold with surface openings (e.g., valves and veins), yet it remains topologically equivalent to a sphere (genus 0), lacking three-dimensional through-holes characteristic of a torus (genus 1) or a double torus (genus 2).

A wavefront is defined as the transition zone between excited and excitable tissue which, as it propagates, leaves a refractory tail that gradually recovers to the excitable state (Wiener and Rosenblueth, 1946; Arno et al., 2024a; b). Consequently, on a two-dimensional surface, this wavefront is a one-dimensional object, which topologically may take one of two fundamental forms: line segments and rings. We term the line-like wavefronts open waves, and the ring-like wavefronts closed waves. Figure 1 shows examples of the two types, with open waves in panel A and closed waves in panel B.

**FIGURE 1:**
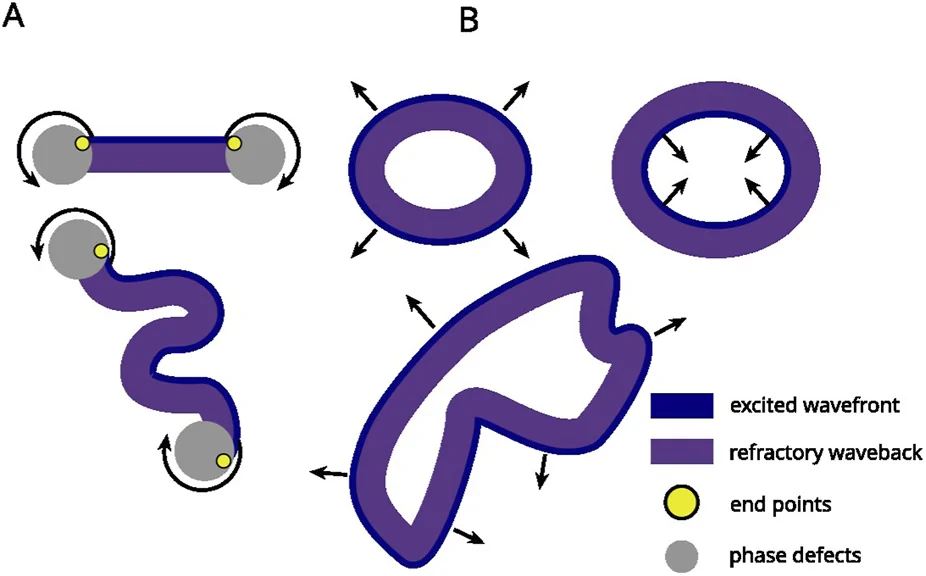
Illustrated examples of the two wave types. **(A)** Open waves are topologically equivalent to line segments, are attached to a phase defect (anatomical obstacles, scar tissue, or functional obstacles like refractory tissue), and are bounded by two endpoints of opposite chirality. **(B)** Closed waves are topologically equivalent to rings, and have no endpoints. Depending on the direction of propagation, a closed wave may expand or shrink.

#### 2.1.1 Open waves

We define an open wave as a wavefront topologically equivalent to a line segment, where each end is coupled to a boundary, either anatomical (e.g., valves, veins) or functional (e.g., refractory tissue) (Arno et al., 2024a; Tomii et al., 2021), see Figure 1A.

Furthermore, we define endpoints as the points wavefront intersect with a boundary, see Figure 1A. Consequently, each open wave contains two endpoints, each attached to a phase defect. These phase defects may correspond to anatomical structures, such as veins and valves; locally inexcitable tissue, such as scar tissue; or excitable tissue that is temporarily in a refractory state (Arno et al., 2021). Each endpoint is assigned a chirality $\left(\xi_{e}=\pm 1\right)$ , depending on whether the surrounding tissue transitions from an excited to a resting state in a clockwise or counterclockwise direction.

Critically, endpoints are distinct from rotation centers (phase singularity), while the latter inherently exist as a property of discontinuous phase defects in excitable media (Arno et al., 2021; Tomii et al., 2021), endpoints only signify the specific connection of a wave to such a defect. Crucially, multiple endpoints can attach to the same phase defect. Therefore, the total rotation index I of a phase defect is calculated as the sum of the chirality of all attached endpoints, mirroring the methodology used to count phase jumps in singularity identification. A phase defect may also coincide with an anatomical boundary. More generally, all phase defects including anatomical boundaries are treated equivalently and will hereafter be denoted by the letter C of circumference. A visual representation of this discrepancy is shown in Figure 2B.

**FIGURE 2:**
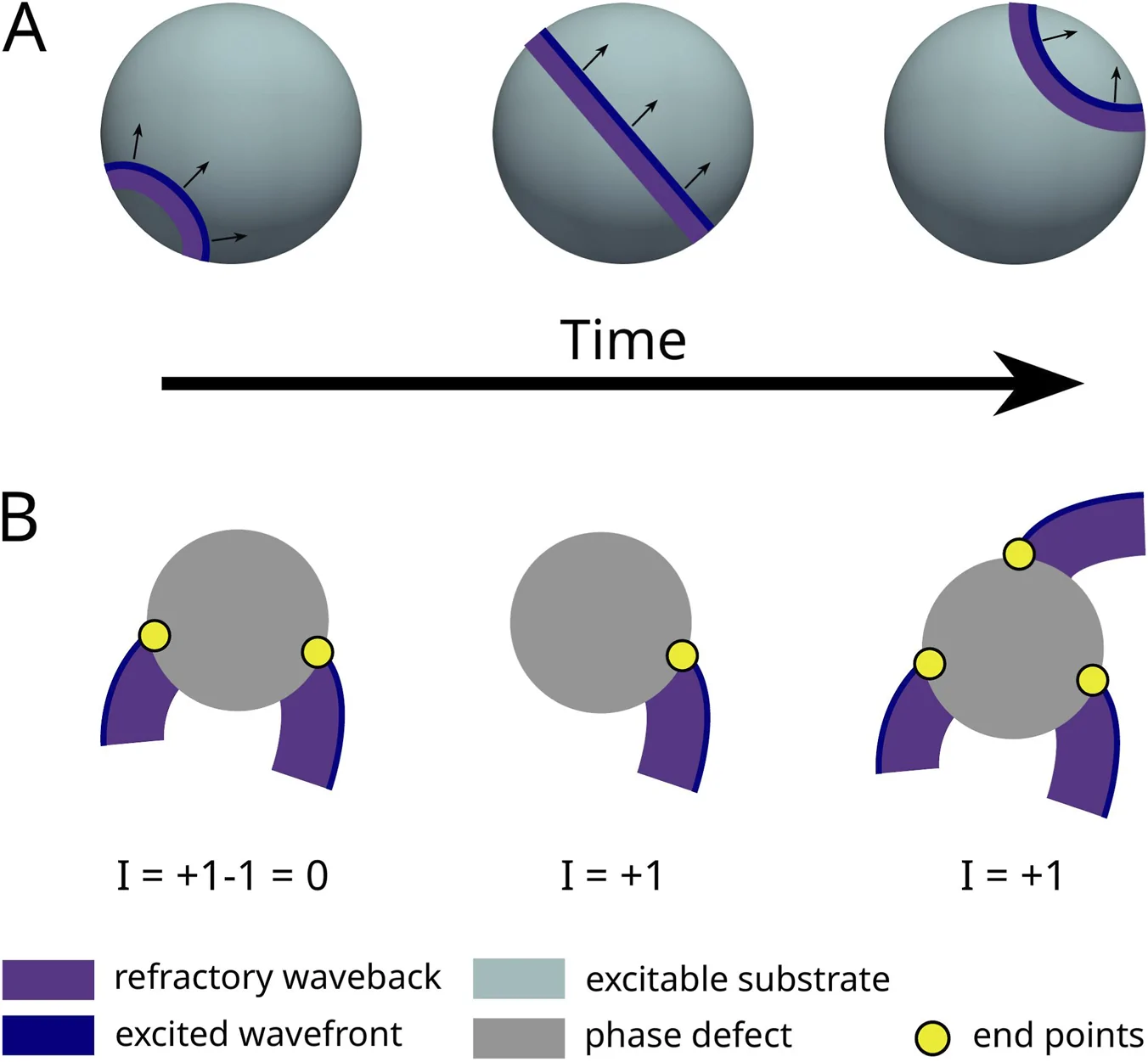
**(A)** Example of the time evolution of a closed wave constrained to a two-dimensional surface embedded in three-dimensional space, in this case a sphere. from left to right, the wave expands to a certain extent, and eventually begins to contract, leading to collapse. **(B)** discrepancy between endpoint chirality and rotation index I . The rotation index is 0 in the left scenario because an equal number of endpoints with opposite chirality are connected to the phase defect. In the center and right panels, an unequal number of points with opposite chirality are connected to the same phase defect, resulting in a non-zero rotation index.

Under this framework, open wave refers universally to any wave possessing a 1D line-segment topology, whereas rotors, wavelets, and anatomical reentries represent specific dynamic manifestations of an open wave defined by its boundary constraints.

- Rotors/Wandering Wavelets: Open waves anchored to functional phase defects, whose time-varying geometry causes the wave tip to meander.
- Anatomical Reentries: Open waves anchored to fixed, non-conducting anatomical boundaries, resulting in stable rotation.

Because the topological structure of the 1D wave segment remains invariant across all these scenarios, “open waves” serve as a unified classification, differing only in the dynamic nature of the phase defects to which they are bound.

In specific cases where both endpoints of an open wave reside on the same phase defect, the endpoints may merge. Depending on the direction of the wavefront, this merger results in either the formation of a closed wave or an annihilation of the wave.

#### 2.1.2 Closed waves

We define a closed wave as a wavefront that forms a closed ring, enclosing one region entirely. Consequently, a closed wave contains no endpoints. Figure 1B illustrates some examples of closed waves.

Unless interrupted by collisions, a closed wave may propagate outward or inward, causing it to either expand or shrink, depending on the relative positions of excited and excitable tissue. However, on a two-dimensional surface of genus 0, each expanding close wave will eventually transition to a shrinking wave and terminate, as shown in Figure 2A.

Closed waves can arise from focal activity, can be induced by external electrical stimuli such as pacing, or emerge spontaneously from wave collisions, as discussed in the following section. The pulses generated by the sino-atrial node also act as closed waves, which expand through the atrium and eventually collapse.

### 2.2 Wavefront interactions

In addition to their behavior in isolation, waves may collide with other objects, such as other waves and anatomical or functional obstacles, altering the propagation pattern. Depending on the substrate geometry and conduction velocity, a wave may also collide with itself. Upon undergoing such an interaction, waves of either category may split, merge, and recombine, giving origin to new waves. The following sections describe the five basic configurations of these interactions, based on the input and output types of each reaction. Any event in which the total number of a wave type increases is considered a creation event, either an open or closed wave creation for that wave type. Any in which the total number decreases is considered an annihilation event, either an open or closed wave annihilation. However, we also present an example involving simultaneous open wave creation and annihilation, preserving the total open wave number: the open wave collision. Most notably, one interaction can host multiple events simultaneously as is the case in Figures 3A,D. Additionally, because of the limited temporal resolution of the simulations, several base interactions may appear as a single larger interaction, potentially containing multiple events.

**FIGURE 3:**
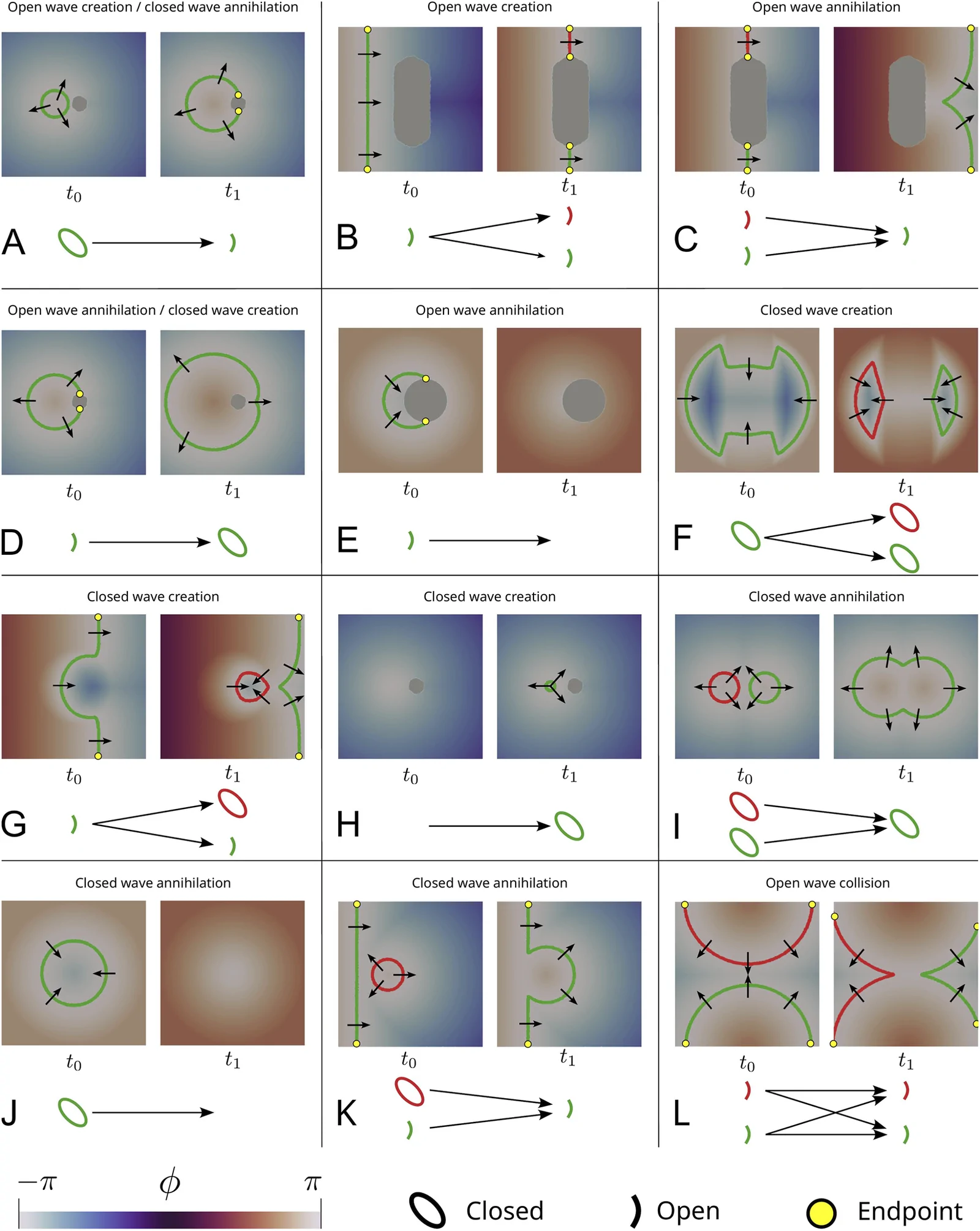
Examples of all interactions according to the theoretical framework. The colors in the panels represent the phase of the simulation, while the red and green lines highlight the wavefronts in the system. Gray represents inexcitable tissue. **(A)** shows an open wave creation and a closed wave annihilation. **(B)** shows an open wave creation from one open wave. **(C)** shows an open wave annihilation from two open waves. **(D)** shows an open wave annihilation with a closed wave creation. **(E)** shows an open wave annihilation. **(F)** shows a closed wave creation from one closed wave. **(G)** shows a closed wave creation from one open wave. **(H)** shows a closed wave creation from focal activity. **(I)** shows a closed wave annihilation from two closed waves. **(J)** shows a closed wave annihilation. **(K)** shows a closed wave annihilation through collision with an open wave. **(L)** shows a open wave collision, with no creation or annihilation.

#### 2.2.1 Event 1: Open wave creation

When a wavefront collides with an obstacle and creates a new pair of endpoints, a new open wave is created. This can occur in two different ways: when a closed wave collides with an obstacle and becomes open, as seen in Figure 3A; or when an open wave collides with an obstacle, splitting into two open waves, as seen in Figure 3B.

#### 2.2.2 Event 2: Open wave annihilation

Conversely, when an interaction causes two opposite endpoints to collide, they disappear, and cause an open wave to be annihilated. This can occur between the endpoints of two distinct open waves, causing them to merge into one open wave, as seen in Figure 3C; or between the endpoints of the same open wave, which becomes closed, as seen in Figure 3D.

The latter case may result in either an expanding or shrinking closed wave, depending on geometry and conduction direction. For certain configurations, the resulting shrinking wave may be very small and disappear quickly. In the limiting case, collapse effectively occurs simultaneously with endpoint collision, without a detectable intermediate stage as a closed wave. This degenerate case is illustrated in Figure 3E.

#### 2.2.3 Event 3: Closed wave creation

Closed waves may be created from the already discussed open-to-closed transition, as seen in Figure 3D. Additionally, Figure 3F shows a closed wave self-collision from a shrinking closed wave, resulting in two smaller closed waves; and Figure 3G shows an open wave self-collision, resulting in an open and a closed wave after the interaction is finished. Finally, a closed wave may be created through focal activity, with no interaction between previously existing waves, as shown in Figure 3H.

#### 2.2.4 Event 4: Closed wave annihilation

Closed waves may be annihilated from the already discussed closed-to-open transition, as seen in Figure 3A. Additionally, when two closed waves collide, they merge into one, as seen in Figure 3I. A closed wave, if uninterrupted by collisions, will eventually collapse into itself, disappearing as shown in Figure 3J. Finally, closed waves can also annihilate when interacting with an open wave as depicted in Figure 3K.

#### 2.2.5 Event 5: Open wave collision

A final type of interaction is the open wave collision. In this type of contact, two open waves collide frontally with one another and produce two new open waves. As the total number and types of waves remains the same, this interaction is not a creation or annihilation event. An example of which can be seen in Figure 3L.

## 3 Methods and materials

### 3.1 Simulation data

Analysis was performed on a previously described dataset of 600 patient-based AF simulations (Bezerra et al., 2025). They were generated using the openCARP simulation software (Plank et al., 2021) from a publicly available dataset of 100 triangulated three-dimensional meshes from AF patients which included realistic fibrosis and fiber orientation information (Roney et al., 2022). The Courtemanche ionic model (Courtemanche et al., 1998) was used with AF remodeling, encompasses a reduction of the ultra-rapid delayed rectifier conductivity $\left(G_{\mathrm{Kur}}\right)$ by 50%, a reduction of transient outward potassium $\left(G_{to}\right)$ by 50% and a reduction of 70% to the L-type calcium conductivity $\left(G_{\mathrm{CaL}}\right)$ . Furthermore, fibrotic regions were identified based on intensity of Late Gadolinium enhanced MRI. These regions were remodeled by reducing $G_{\mathrm{Kur}}$ by 50%, $G_{to}$ by 50%, $G_{\mathrm{CaL}}$ by 50% and $G_{Na}$ by 30%. Additionally, the conduction velocity was lowered linearly with the MRI-intensity from 0.81 mm/ms to 0.53 mm/ms.

For each mesh, six different starting conditions were simulated, where each time, a phase singularity with clockwise or counter-clockwise direction was imposed on the origin of one of the three Cartesian planes of the three-dimensional space. Each simulation lasted 3400 m each. Transmembrane voltages were recorded at a sampling frequency of 200 Hz to save on storage space and computation time. For a full description, we refer to our previous AF work (Bezerra et al., 2025).

### 3.2 Open and closed wave detection

To identify wavefronts, we used an extended version (Verstraeten et al., 2026) of established phase mapping methods (Umapathy et al., 2010; Lootens et al., 2024) that can be found in the open-source repository: https://gitlab.com/opendgm/opendgm. The algorithm uses this framework to identify all open and closed wave. The code of which is stored in this Gitlab repository: https://gitlab.com/opendgm/paper-af-open-and-closed-waves.

First, the phase (ϕ) values were calculated from the transmembrane voltage of each mesh vertex using the Sawtooth method, which linearly interpolates the phase of a point between subsequent LATs.

Second and most importantly, the extended phase mapping method includes the concept of “phase defects”, which are discontinuities in the phase-field (Arno et al., 2024a; Tomii et al., 2021), caused by functional block and fibrosis. These defects break the continuity requirement of the rotation calculation Equation 2 and are therefore removed from the analysis. In order to identify defects at each time-step, the phase differences Δϕ on each triangle-edge were calculated, and when 0.2π<|Δϕ|<1.8π (Arno et al., 2024a; Tomii et al., 2021), the corresponding edge was marked as discontinuous and the related triangles were identified as phase defects (Figure 4, gray) and excluded from the wavefront detection.

**FIGURE 4:**
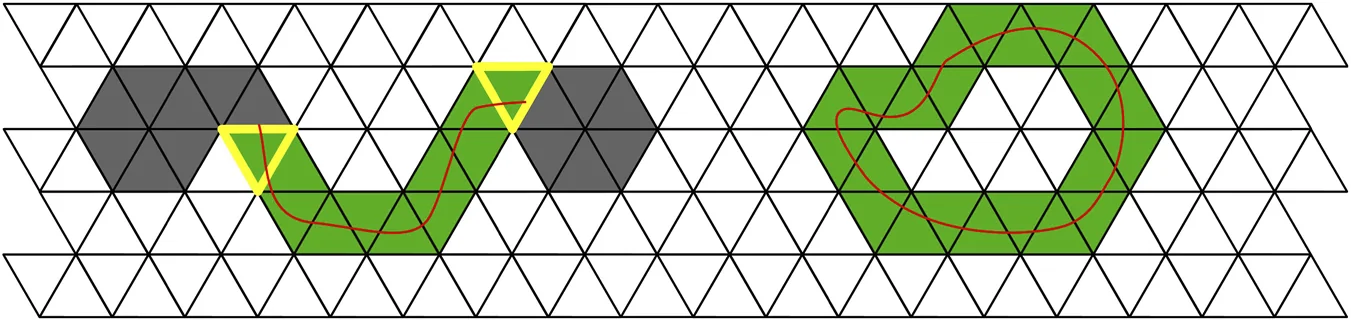
Wavefront identification using the extended phasemapping method on a triangulated mesh. The underlying wavefront of the system is presented in red. First, the phase defects are identified in gray, then the wavefront triangles are identified in green and clustered by means of shared edges that contain a phase jump. End-triangles are identified at the intersection of these jump-bearing edges and phase defects. In this system, One open wave and one closed wave are detected on the left and right, respectively.

In the resulting phase-field, triangles with 2 phase jumps in opposite direction (Umapathy et al., 2010) (|Δϕ|≥1.8π) , were identified as part of a wavefront (Figure 4, green). These triangles were clustered into connected components, based on a created network of shared edges that contain a phase jump. As such, each connected component represented an individual wavefront. For example, in Figure 4, we have 2 separate wavefronts.

From these wavefront components, we identified end-triangles, which serve as discrete, finite-sized equivalents of endpoints. We can identify an end-triangle as a triangle when one of its edges containing a phase jump is part of a phase defect, signaling the end of the wavefront. Each end-triangle is assigned a chirality value of ±1 , calculated by summing the signs of its edges’ phase jumps, excluding any jumps located at boundary edges, such as anatomical openings and phase defects. Wavefronts were tagged as open waves when they included exactly two end-triangles with opposite chirality, and tagged as closed waves when they had no end-triangles.

### 3.3 Wavefront events

Wavefronts were tracked over time using a nearest-neighbor matching algorithm. First, for each point in a wavefront at time t , the closest wavefront point at time t−1 was identified (within a distance of 5 mm). Each time a new wavefront at time t−1 was reached in this way, a backward link was created. This was repeated for each wavefront and each time step, thereby compiling a network of connections between different wavefronts, propagating backwards in time. This network was finally reversed to represent the proper wavefront dynamics of each simulation.

From this graph, we extracted all wavefront interactions from the graph by removing connecting arrows between open and closed waves that do not undergo any interaction but simply propagate through space unchanged. This reduction entails the removal of incoming arrows to nodes with in-degree equal to one and outgoing arrows from nodes with out-degree equal to one, if the arrows preserve wave types between start and end point. The resulting filtered graph is a collection of subgraphs on which we apply a connected-components filter, where each separate subgraph represents a wavefront interaction between two time-steps.

Most notably, these final interactions (the subgraphs) can be combinations of different base-events, and can contain multiple events in one. We can identify the separate five events by extracting the difference in wave type count from t and t−1 to obtain the number of creation (Event one and 3) and annihilation events (Event two and 4) for each wave type.

$${\text{Open wave creation}}_{t}=\text{max }\left(0,{\text{Open waves}}_{t}-{\text{Open waves}}_{t-1}\right){\text{Open wave annihilation}}_{t}=\text{max }\left(0,{\text{Open waves}}_{t-1}-{\text{Open waves}}_{t}\right){\text{Closed wave creation}}_{t}=\text{max }\left(0,{\text{Closed waves}}_{t}-{\text{Closed waves}}_{t-1}\right){\text{Closed wave annihilation}}_{t}=\text{max }\left(0,{\text{Closed waves}}_{t-1}-{\text{Closed waves}}_{t}\right)$$

Additionally, the open wave collisions (Event 5) were obtained by counting the open waves that have an out-degree >1 at time t−1 minus 1. Let O(t−1) be the set of open waves at time t−1 , and let $d^{+}\left(i,t-1\right)$ denote the out-degree of wave i∈O(t−1) . The number of collision events (Open wave ${\text{collision}}_{t}$ ) at time t is then

$${\text{Open wave collision}}_{t}\;=\;\left(\underset{i\;\in \;O\left(t-1\right)}{\sum }1\left[d^{+}\left(i,t-1\right)>1\right]\right)-1$$

where 1[⋅] is the indicator function (1 if the condition holds, 0 otherwise).

### 3.4 Index calculation

To validate the wavefront detection analysis, we calculated the phase index within each episode (Winfree and Tyson, 1988; Bray and Wikswo, 2002). This value I represents rotational activity around a certain area S with boundary C .

However, in our simulations, the endpoints do not directly correspond to rotation. Instead the index of rotation I is determined by the sum of the endpoint chirality $\left(\xi_{e}\right)$ of all attached endpoints e to the phase defect C as described in Equation 1.

$$I=\underset{e}{\sum }\xi_{e}.$$ (1)

This method is equivalent to calculating phase singularities by summing the phase jumps using conventional phasemapping, using Equation 2:

$$I=\frac{1}{2\pi }\underset{C}{\oint }\vec{\nabla }\phi \cdot d\vec{l},$$ (2)

where C is the path that this integral is following, $\vec{\nabla }\phi$ is the spatial gradient of the phase value at the current time, and $d\vec{l}$ is the direction that follows the tangent to the curve C .

Additionally, recent research emphasizes the importance of incorporating anatomical structures and functional defects into phase maps to obtain a more complete and accurate representation of the underlying dynamics (Arno et al., 2021; Verstraeten et al., 2026; Van Den Abeele et al., 2025; Duytschaever et al., 2024; 2025). Accordingly, the phase index is computed for each phase defect in the system to identify all local rotations. These contributions are then summed to obtain the total index, which, according to the index theorem (Davidsen et al., 2004), must vanish on compact surfaces, as considered here.

### 3.5 Virtual ablation

Compared to our previous research, the ablation strategy was re-evaluated to fit within our new theoretical framework. The aim of this new strategy is to transform all open waves into shrinking waves whose endpoints adhere to a common boundary. This configuration enables annihilation when its endpoints approach each other, analogous to the novel teleportation mechanism in DeTal et al. (2022) in which open-wave endpoints are teleported through addition of extra stimuli to eliminate rotations caused by open waves.

To evaluate the impact of ablation timing, the baseline simulation was run up to a total duration of 3400 m. At three distinct time points: 1000 m, 2000 m, and 3000 m post-simulation onset—separate simulation branches were initialized. In each independent branch, instantaneous virtual ablation lines were introduced. For each specific branch, the exact ablation lines were determined using the extended phase mapping algorithm from the openDGM framework to detect all phase defects as described in the previous section (Figure 5 left panel). These defects were then added as primary ablation targets.

**FIGURE 5:**
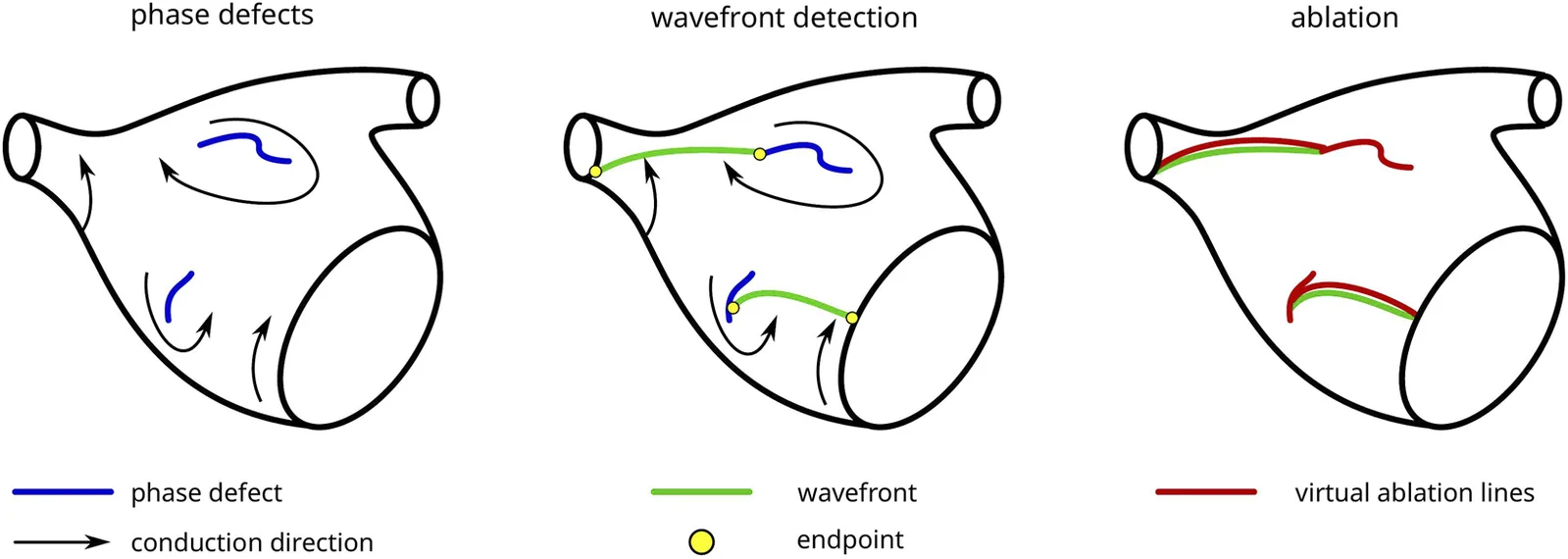
Virtual ablation pipeline. On the left panel, the phase defects are identified. Then, on the center panel, the open wavefronts are detected. Finally, in the right panel, instantaneous ablation lines are applied in the simulation.

The second part of the virtual ablation was directed at the open waves (Figure 5 center panel), in which endpoint pairs of the same wave were connected immediately ahead of the activation wavefront. This procedure ensures that endpoints would be connected to the same obstacle. To create this virtual ablation line, the A* traversal algorithm was used to find the lowest-cost path between the two points. The edge weights of the adjacency matrix were defined using a heuristic distance function, $d_{\mathrm{heur}}$ (Equation 3), designed to preferentially guide paths through regions of low phase while simultaneously minimizing ablation length. The heuristic combined the local phase values ϕ at neighboring points with a positive offset to ensure strictly positive weights:

$$d_{\mathrm{heur}}\left(i,j\right)=\left(1.1\pi -\phi \left(i\right)\right)\left(1.1\pi -\phi \left(j\right)\right)$$ (3)

where ϕ(i) and ϕ(j) denote the phase values at neighboring spatial locations i and j , respectively. Lower phase regions therefore corresponded to lower traversal costs, encouraging ablation paths to align with areas more likely to intercept active wave propagation.

Both targets (phase defects and open waves) were joined into one instantaneous virtual ablation (Figure 5 right panel). After which, each simulation was analyzed for termination by detecting activation times. If activation stops within the 400 m window, then the ablation was determined a success. Otherwise the ablation was labeled a failure.

## 4 Results

### 4.1 Open and closed waves detection

In total in all simulations, 804,085 open waves were detected, of which 185,877 where its end-points are located at the same boundary. Additionally, 51,039 closed waves were detected. Notably, all open waves contain exactly two endpoints with opposite chirality and all closed waves 0 endpoints. Illustrating this result, five representative examples will be shown in the subsequent section.

### 4.2 Wavefront events

Over the span of 600 AF simulations, a total of 132,870 subgraphs were found, containing 274,461 events, achieving an average of 2.07 events per subgraph. Of these events 45% were creation events (Event one and 3) and 45% were annihilation events (Event two and 4). 77% of events involved a change in open waves (Event one and 2) and 13% a change in closed waves (Event three and 4). Finally, 9% of the subgraphs were found to be open wave collisions (Event 5). An overview of which can be found in Table 1.

**TABLE 1:** Summary of event categories. A majority of the subgraphs (86%) are dependent on open waves.

Interaction	Open waves	Closed waves	Total
Creation	39%	7%	45%
Annihilation	39%	7%	45%
Collision	9%	N/A	9%
Total	86%	13%	100%

To illustrate the complexity of these wave-interactions, representative examples are shown in Figure 7. These episodes range from simple, semi-periodic dynamics to highly complex simulations featuring multiple rotational centers and wave breakup. This difference in complexity is directly reflected in the corresponding graph representations (constructed as detailed in Section 3.2). Specifically, simpler simulations exhibit fewer events, as shown in Figure 6A,B (snapshots of the same simple case), while more complex dynamics yield denser graphs, as seen in panels C and D (snapshots of the same complex case).

**FIGURE 6:**
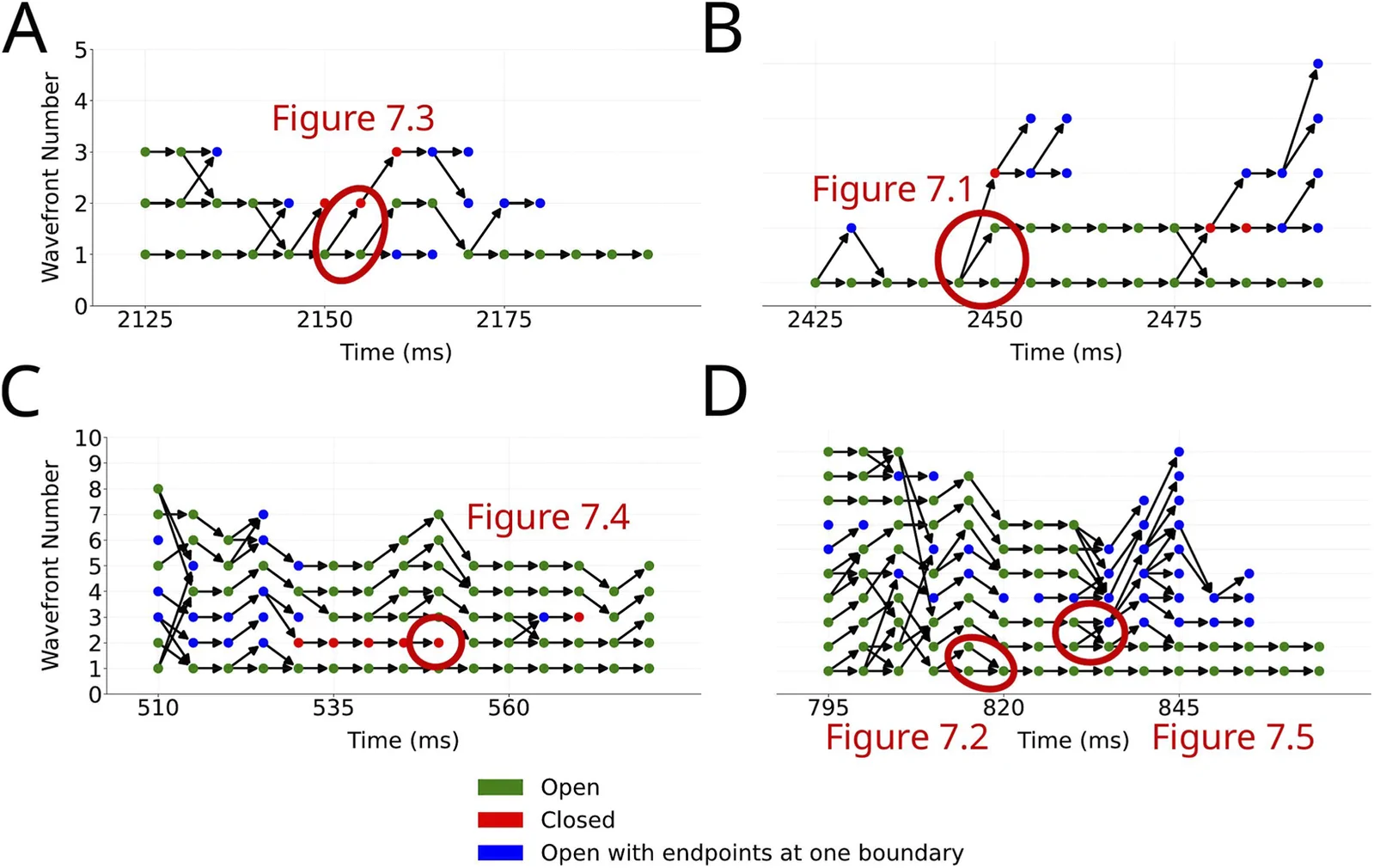
Graph representations of wave dynamics for a simple episode, with one rotor and one anatomical reentry from 2125 m to 2195 m in panel **(A)** and from 2425 m to 2495 m in panel **(B)**. Interactions of a more chaotic episode, containing up to six simultaneous rotations in total, are found in panel **(C)** from 510 m to 580 m and **(D)** from 795ms to 865 m.

During a 500 m window, the simple case contains a total of 62 wave events: eight closed wave creations, six closed wave annihilations, 22 open wave creations, 22 open wave annihilations, and four open wave collisions. In the more complex episode (Figures 6C,D), a total of 212 wave events were identified in a similar 500 m window: four closed wave creations, four closed wave annihilations, 93 open wave creations, 90 open wave annihilations, and 21 open wave collisions.

### 4.3 Representative examples

To analyze the identification of open and closed wave configurations, we consider specific case studies depicted in Figure 7. These example snapshots are schematically illustrated in Figure 6, obtained from the wave tracking algorithm in Section 3.2.

**FIGURE 7:**
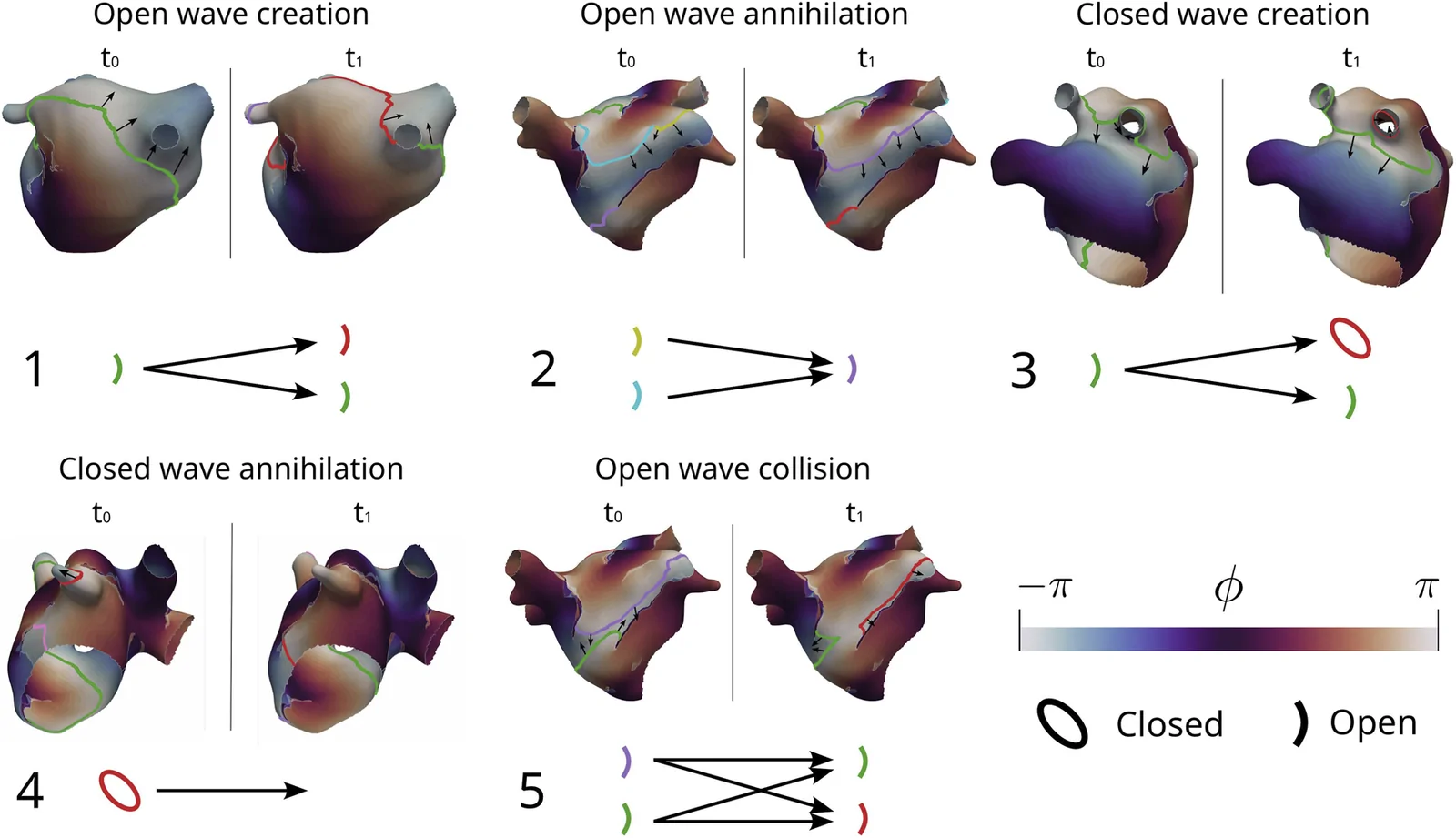
Representative examples in realistic AF simulations for each type of event. Each example shows an snapshot of the phasemap from before $\left(t_{0}\right)$ and after the event $\left(t_{1}\right)$ . Underneath each scenario, a color-coded graph representation is presented, representing the isolated event in each scenario. (1) shows an open wave creation through collision of an open wave (green) with the right inferior pulmonary vein, splitting the wave in two open waves (red and green). This is represented in Figure 6B and is an example of Figure 3B. (2) shows two open waves (yellow and blue), separated by a small phase defect, that merge into one open wave (purple). This is represented in Figure 6D and is an example of Figure 3C. (3) shows an open wave (green) that collides with itself, creating an additional closed wave in the process (red). This is represented in Figure 6A and is an example of Figure 3G. (4) shows a closed wave shrinking until it disappears. This is represented in Figure 6C and is an example of Figure 3J. (5) shows two open waves (purple and green) colliding head-on and creating two new open waves (green and red). This is represented in Figure 6D and is an example of Figure 3L.

#### 4.3.1 Open wave creation

In the simple case, at the $t_{0}=2445\;ms$ , a single open wave is present, with endpoints at a posterior phase defect and the mitral valve (see Figure 6B for the graph representation; Figure 7 for a visualization of the wave). At the subsequent time point $t_{1}=2450\;ms$ , three distinct waves are observed: an open wave extending from the mitral valve to the right inferior pulmonary vein, a second open wave connecting the right inferior pulmonary vein to the posterior phase defect, and a closed wavefront encircling the left inferior pulmonary vein. The highlighted base-event in Figure 6B and the corresponding view at $t_{1}$ in Figure 7 illustrate how the initial open wave (green) splits upon encountering the right inferior pulmonary vein, resulting in two open waves (green and red) at $t_{1}$ . This is an example of Figure 3B.

#### 4.3.2 Open wave annihilation

The subgraph in Figure 6D contains a total of nine different open waves at $t_{0}=815$ ms. At $t_{1}=820$ ms two pairs of open waves have combined into other open waves, resulting in a total of seven open waves in the system, with endpoints, connecting to various phase defects and anatomical objects. In Figure 7, we show the highlighted event of the subgraph in Figure 6D: two open waves (light blue and yellow) separated by a phase defect at $t_{0}$ merge into a single wave (purple). This is an example of Figure 3C.

#### 4.3.3 Closed wave creation

The subgraph in Figures 6A, 7 shows an open wave (green) at $t_{0}=2150\;ms$ , connecting the mitral valve to a posterior phase defect, additionally a closed wave is present at the anterior wall, which is visible in Figure 6, but not in Figure 7. Looking at the interaction of the open wave, at $t_{1}=2155\;ms$ , the open wave encounters the left inferior pulmonary vein, leading to a self-collision at $t_{1}$ . As a result, the open wave (green) continues along its original trajectory, while a new closed wave (red) is created around the vein, see right panel Figure 7. This is an example of Figure 3G.

#### 4.3.4 Closed wave annihilation

At $t_{0}=550$ ms, the subgraph at Figure 7 depicts the initial state, comprised of seven wavefronts: six open waves with endpoints linked to two distinct phase defects or anatomical structures, and one closed wavefront that enfolds the left atrial appendage. This closed wavefront is shown in red in the left panel of Figure 7. At time $t_{1}$ , this wave undergoes self-annihilation disappearing from the tissue, see the right panel of Figure 7 and the event at Figure 7. This is an example of Figure 3J.

#### 4.3.5 Open wave collision

For the subgraph shown in Figure 6D, at $t_{0}=830$ ms the simulation contains a total of seven open waves, one of which has its endpoints at the same boundary. In the next timestep, $t_{1}=835$ ms, the simulation retains six of the seven open waves, but now four open wave have endpoints at the same boundary. In the isolated event shown in Figure 6D and Figure Figure 7 two open waves (purple and green) collide frontally, resulting in the generation of two new open waves (green and red) at $t_{1}$ . This is an example of Figure 3L.

### 4.4 Index calculation

Using the novel method described in Section 3.3 that analyzes the rotational activity using phase defect, the positive and negative rotational index of each simulation was tracked over time. One such example is shown in Figure 8, where the positive and negative indexes overlap during the entire simulation, adding up to a consistent total rotational index of 0, consistent with the index theorem. When calculating the index for each of the 600 simulations, we find that at each time the total index is 0.

**FIGURE 8:**
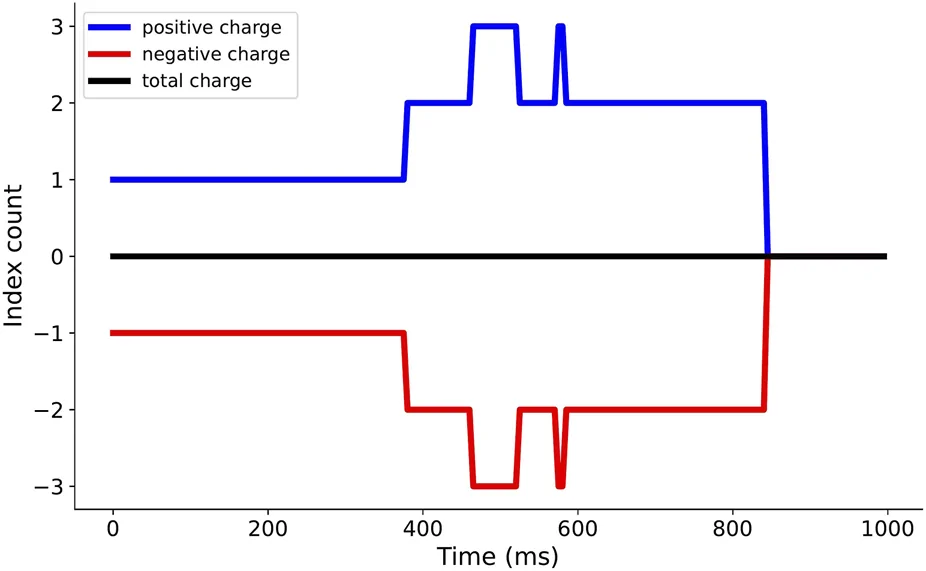
Time series of the positive and negative rotation index in on simulation. Positive index can be seen in blue and negative index in red, with the sum of the indexes in black. The total index of the system remains 0 for each point in time. Video material of the phase map and its positive and negative phase rotations can be found here: https://youtu.be/eOeNesdfKTc.

### 4.5 Virtual ablation

At 1000 m, 2000 m, and 3000 m post-simulation onset, separate simulation branches were initiated, provided active excitation was still present at those times. Within each independent branch, instantaneous virtual ablation was performed. In total, 1475 ablation procedures were executed. Each procedure adding an ablation for each phase defect and each open wave in the system at time of ablation.

In 100%of performed virtual ablations, all wavefronts annihilated, creating electrical silence before the end of the simulation time. An average virtual ablation required 3.12 open waves to be ablated. A median ablation procedure required two ablation lines. An example case is shown in Figure 9, where the ablation of the endpoints interconnected the critical phase defect on the anterior of the left atrium to the MV, and from the MV to a second phase defect on the posterior side of the septum. This virtual procedure with only two ablation lines had termination as the result, as was the case with all other ablations.

**FIGURE 9:**
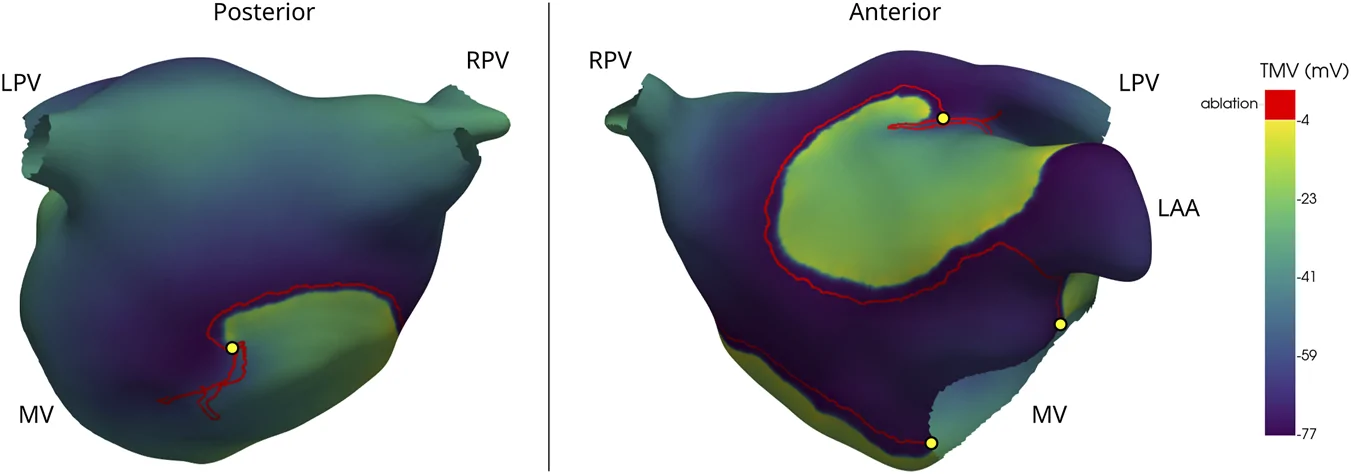
Ablation of atrial activity in the left atrium for a simulation, showing transmembrane voltage at 3000 m. At the time of detection, the algorithm identified one phase defect on the anterior and one on the posterior wall. The path of the virtual ablation (Red) was determined to connect each of its phase defects to the mitral valve (MV) by interconnecting the endpoints (Yellow) of the open wavefronts.

## 5 Discussion

### 5.1 A novel theoretical topological framework

Building upon prior work on atrial topology, this study establishes that complex fibrillatory patterns emerge from a minimal framework comprising two wave types (open and closed) and five fundamental wave events: creation and annihilation of both wave types alongside open wave collision. Analysis of 600 patient-based atrial fibrillation simulations revealed all wave interactions conform to this model, either individually as described in Section 2 or through combinations of multiple events.

#### 5.1.1 Open and closed waves

As shown in Section 4.1, open waves were predominant in our dataset. This is consistent with our simulation design, which was configured to be rotation-driven (see Section 3). Consequently, the presence of at least one open wave is necessary to sustain the AF episode. More complex episodes with more rotational activity have more waves in general, compared to simple cases.

#### 5.1.2 Wavefront events

The graph-based method provides a consistent framework for characterizing wavefront evolution. It is able to consistently identify the five core mechanisms: (1) open wave creation, (2) open wave annihilation, (3) closed wave creation, (4) closed wave annihilation and (5) open wave collision. More complex interactions, represented by larger subgraphs, are always composed of combinations of these five fundamental events.

#### 5.1.3 Index theorem

When evaluating adherence to the Index Theorem within the framework established in Bezerra et al. (2025), our current approach yields superior results. Unlike prior work, which employed clustering of endpoints, thereby suffering from artifactual errors in index conservation, our method utilizes phase defects to achieve perfect adherence to the theorem.

#### 5.1.4 Virtual ablation

Most notably, due to the improved accuracy of endpoint detection with phase defects, the newly revised ablation strategy achieved a 100% success rate in terminating the simulated AF. This absolute termination of rotational activity highlights the effectiveness of our approach and positions it as a robust candidate for future translational research in AF ablation.

### 5.2 Prior applications of topology to AF

Many of the topological concepts this work uses were further developments on previous works in the literature, which we sought to simplify, while trying to avoid some of their limitations.

Wiener and Rosenblueth (1946) were, to our knowledge, the first to identify the categories of open and closed waves. They did so in the context of atrial flutter with constant and isotropic conduction velocity, and identical refractory periods through the entire tissue. They stated, in the form of a theorem, that if at least one refractory period has elapsed since the last stimulus, all waves present in two-dimensional tissue would be “either an open or a closed path”. They also observed that if a wave was open, its endpoints would necessarily either lay at the geometric boundaries of the tissue, or at refractory tissue. From these basic mathematical assumptions, the existence of open and closed waves was deduced as a notable consequence. However, the authors did not explore further implications such as the self-propagating and self-terminating tendencies of each wave type, and did not to extend the framework to AF.

Marcotte and Grigoriev (2017) demonstrated that wavelets terminate at pairs of oppositely charged singularities and analyzed transitions between wave states using four phase-map level sets. Their framework provides a topological description of excitation based on the dynamics of wavefronts, wavebacks, and the point defects that anchor wavelets. Compared with our framework, the authors added additional level-sets (wavebacks), which is not essential from a theoretical standpoint in this work. Importantly, their formulation does not include a direct analogue of closed waves.

Most closely related to this research, is the work by DeTal et al. (2022). Here, the authors use concepts analogous to those of our open and closed waves, but all of the mechanisms they describe neatly fit into our framework. DeTal et al. (2022) used properties of wave topology to propose a “teleportation” method of localized defibrillation by generating localized pulses to terminate arrhythmia. The teleportation method consists of destroying a singularity and creating a new one somewhere else, reshaping the targeted spiral wave. When two singularities are “teleported” together, they are forcing an open wave annihilation, which can be repeated for all pairs of endpoints to close all waves. In a similar way, we are inducing ablation lines with the aim to teleport the endpoints to one-another to promote open wave annihilation.

Arno et al. (2021), Arno et al. (2024a) identified limitations of phase singularities in the presence of phase discontinuities and boundaries, subsequently developing a quasi-particle framework analogous to particle physics that retains analytical tractability. This approach characterizes bound states (e.g., head and tail quasi-particles that coalesce into classical phase singularities), employs Feynman-like diagrams to model temporal evolution of quasi-particle interactions, and describes dynamics of wavefronts, wavebacks, and phase defects. Our framework maintains consistency with their Feynman-like diagrams and generalizes their wave interaction taxonomy, but Arno’s work lacks quasi-particles for closed wavefronts—an absence stemming from its focus on linear-core rotors—limiting its applicability to the full spectrum of clinically relevant AF behavior.

Furthermore, Ganesan et al. (2025) proposed a topological approach to unify various theories of atrial arrhythmias through the lens of statistical physics. The authors draw parallels with the Kosterlitz-Thouless (KT) transition in two-dimensional systems, where the interplay of vortex-antivortex pairs delineates distinct physical phases. Their hypothesis suggests that AF emerges from a disordered, unbound vortex state akin to the KT unbound phase, while AT presents as an ordered bound vortex state with regular circuitry reminiscent of the KT bound vortex scenario. Similar to our research, the authors utilize topological concepts to unify diverse mechanisms into one cohesive framework.

Our work on atrial topology began with an analysis of the index theorem for AT (Duytschaever et al., 2024; Van Den Abeele et al., 2025; Duytschaever et al., 2025), which we directly translated into a novel ablation strategy: reentries form paired objects that must be targeted jointly for ablation. In subsequent work, we showed that reentries are also paired in AF (Bezerra et al., 2025), using the same simulation set as in this study. However, we found that simply connecting opposite singularities in AF is not always sufficient to terminate reentry, which directly motivated the present work.

### 5.3 Implications for the treatment of AF

The proposed fundamental processes of wavefront creation and annihilation offer a straightforward description of the mechanisms underlying arrhythmia ablation. Furthermore, this framework can be utilized to evaluate ablation efficacy, expanding upon our previous work in AT (Duytschaever et al., 2024; Van Den Abeele et al., 2025; Duytschaever et al., 2025) and AF (Bezerra et al., 2025). In our prior AT studies, the recommended strategy involved connecting unexcitable conduction boundaries with a +1 topological index to those with a −1 index. This intervention forces the respective open waves to coalesce into closed loops. Because the remaining system consists exclusively of closed waves or open waves with both endpoints anchored to the same boundary, all wavefronts eventually undergo self-annihilation, successfully terminating the arrhythmia.

Considering AF, it is not guaranteed that the rotations are stable as is the case for AT. The new heuristic ablation offers an extension on the conventional ablation that interconnects boundaries by blocking the wavefront path directly. However, when considering the aggregate of each ablation section in between phase defects, the heuristic strategy remains consistent with the ablation of connecting +1 to −1 rotation centers as discussed previously. For example, in Figure 9, a single ablation from the anterior phase defect to the posterior defect would have the same effect topologically.

While the proposed ablation strategy was effective in each virtual procedure, it is not practically implementable, as it requires the instantaneous creation of a continuous ablation line that precisely blocks the trajectory of a dynamically evolving wave. In addition, we did not perform tests to assess reinducibility, which is a critical factor for evaluating long-term ablation outcomes. We do not expect these cases to be non-inducible, since the intervention was designed only to terminate AF instantaneously. Given that open waves propagate through space and time, there is no reason to assume that AF would not re-emerge upon new induction. Therefore, for real-world application, an alternative strategy is required: in particular, approaches aimed at minimizing the formation of new open waves may represent a promising preventive direction. One practical approach could be to use the wave-detection algorithm in a setting of burst pacing, in combination with high-density local mapping. This method would allow identification of substrate which is prone to generating non-recombining open waves, marking these areas as primary ablation targets. However, it remains to be seen if this approach could work in clinical setting.

### 5.4 Limitations

As this study uses the same dataset as our previous work, it inherits several of its limitations. While it integrates commonly proposed AF mechanisms, including anatomical reentries, rotors, and meandering wavelets, it does not encompass all reported mechanisms of AF. In particular, focal activity, epi–endo dissociation, and bi-atrial arrhythmias were not considered. For focal activity, we do not expect this to alter the theoretical framework. However, epi–endo dissociation and more complex bi-atrial geometries violate the assumption of a two-dimensional embedded surface, and therefore require further methodological development.

### 5.5 Conclusion

This work establishes an intuitive classification of atrial conduction waves into open and closed types, characterizing their distinct properties, interactions, and mutual transformations. By providing a robust methodology for identifying these wave behaviors, the framework offers both a practical detection tool with the theoretical foundation required to understand the mechanisms underlying arrhythmia termination through open-to-closed transitions.

## Data Availability

The scripts for generating the simulation datasets can be found in the following repository: https://gitlab.com/rwvdnabe/paper_open_closed_simulations. The scripts for analysis can be found in following repository: https://gitlab.com/opendgm/paper-af-open-and-closed-waves.
